# In-Situ Assembly of MoS_2_ Nanostructures on EHD-Printed Microscale PVDF Fibrous Films for Potential Energy Storage Applications

**DOI:** 10.3390/polym14235250

**Published:** 2022-12-01

**Authors:** Bing Zhang, Shikang Li, M. Shafin. H. Qureshi, Ukil Mia, Zhenghui Ge, Aiping Song

**Affiliations:** 1 College of Mechanical Engineering, Yangzhou University, Yangzhou 225127, China; 2 State Key Laboratory for Manufacturing Systems Engineering, Xi’an Jiaotong University, Xi’an 710049, China

**Keywords:** electrohydrodynamic printing, MoS_2_-PVDF electrodes, hierarchical structures, energy storage

## Abstract

Three-dimensional (3D) printing has been widely utilized to fabricate free-standing electrodes in energy-related fields. In terms of fabrication, the two most challenging limitations of 3D printed electrodes are the poor printing resolution and simple structural dimension. Here we proposed a novel process to fabricate molybdenum disulfide-polyvinylidene fluoride (MoS_2_-PVDF) hierarchical electrodes for energy storage applications. The 20-layer microscale PVDF films with a stable fiber width of 8.3 ± 1.2 μm were fabricated by using electrohydrodynamic (EHD) printing. MoS_2_ nanostructures were synthesized and assembled on the microscale PVDF fibers by using hydrothermal crystal growth. The structural and material investigations were conducted to demonstrate the geometrical morphology and materials component of the composite structure. The electrochemical measurements indicated that the MoS_2_-PVDF electrodes exhibited the typical charge-discharge performance with a mass specific capacitance of 60.2 ± 4.5 F/g. The proposed method offers a facile and scalable approach for the fabrication of high-resolution electrodes, which might be further developed with enhanced specific capacitance in energy storage fields.

## 1. Introduction

Energy techniques have always been scientific as well as industrial innovations to promote the progress of human civilization. Electrochemical energy storage devices like supercapacitors [1] and lithium-ion batteries [2] are attracting extensive attentions in energy fields. Supercapacitors possess the advantage of fast charging and discharging, long cycling life and desirable safety, which have been widely used in new energy vehicles, flexible electronics, and micromechanical devices [3,4]. The electrodes of supercapacitors should have a large specific surface area, high structural conductivity, and controllable porosity [5,6]. These structural properties significantly determine the electrolyte penetration or electron migration during the electrochemical energy storage process [7]. In order to improve the energy storage performance, various materials synthesis approaches have been explored for the development of new energy storage materials [8,9]. In general, conventional chemical synthesis methods can only optimize the energy storage property by adjusting the materials component or molecular structure, which cannot directly create the electrode structure for energy device applications.

Advanced manufacturing techniques like 3D printing are giving new birth to the fabrication of multiple electrodes in energy storage fields [10,11]. The main advantages of 3D printing like versatile materials options, the direct formation of complex structures, and low costs make it possible to fabricate electrode architectures in a layer-by-layer manner [12,13]. The most prevalent 3D printing technique employed in energy storage fields is extrusion-based 3D printing. For example, Zhou et al. fabricated microscale 3D electrodes with superior shape fidelity and geometric accuracy via extrusion-based 3D printing, which improved surface area accessibility and ion transport efficiency [14]. The 3D-printed self-standing electrode delivered an areal capacitance of 2.02 F/cm^2^ and maintained a capacitance retention rate of 85% after 5000 cycles. The main limitation of extrusion-based 3D printing in energy-related fields is the relatively low resolution of larger than dozens of microns [11]. Derived from conventional electrospinning [15], electrohydrodynamic (EHD) printing has been developed as a mature technique to create micro/nanoscale fibrillar architectures with the fiber width smaller than 1 μm. In our previous study, EHD printing was developed to fabricate sub-microscale fibers with improved cell adhesion capacity [16]. The improved structural features can provide a facilitated electrochemical performance for energy storage. As such, EHD printing might provide a practical approach for the printing of high-resolution electrodes [17].

Due to the decent processing capacity, polyvinylidene fluoride (PVDF) has been widely employed as the printing materials for EHD printing. In addition, PVDF enables superior structural stability and flexibility, which can endure the hydrothermal environment for further modification. Thus, PVDF was selected as the basic material for EHD printing. Molybdenum disulfide (MoS_2_) has confirmed electrochemical capacitance and can be easily fabricated in the form of nanoplates, nanoflowers, and nanosphere. The various forms of MoS_2_ make it possible to enrich and modify the structural features of energy storage electrodes. In this work, EHD printing was employed to fabricate microscale polyvinylidene fluoride (PVDF) fibrous films that were further utilized as the substrates for molybdenum disulfide (MoS_2_) assembly. It was demonstrated that the MoS_2_ nanostructures can be modified onto the PVDF fibers via the hydrothermal crystal synthesis process. The structural and material characterization were performed to investigate the geometrical morphology and materials component of the MoS_2_-PVDF hierarchical architecture. Electrochemical measurements were conducted to explore the potential energy storage capacity as supercapacitor electrodes. The proposed nanostructure assembled film significantly enhanced the surface area of EHD-printed fibers, providing a facile approach for the optimization of energy storage performance by adjusting the printing or assembling parameters.

## 2. Materials and Methods

### 2.1. Materials for EHD Printing

PVDF with a molecular weight of 534,000 was dissolved into dimethyl sulfoxide (DMSO) and acetone. Capstone FS-66 was also dissolved to tune the viscosity and printability of the ink. The weight percent of PVDF and Capstone FS-66 were 18% and 4.2%, respectively. The volume ratio of DMSO and acetone was 1:1. To prepare the printing ink, 0.9 g PVDF and 0.21 g Capstone FS-66 powders were dispersed into 2.5 mL DMSO and 2.5 mL acetone, then stirred for at least 12 h under room temperature and 1 atm pressure. The as-prepared solution was loaded into the syringe and employed as the ink for EHD printing.

### 2.2. EHD Printing of the PVDF Film

A homemade EHD printing platform was developed and employed to print the PVDF film [16]. The commercial medical needle (23G, Shanghai Ducheng Electronic Co. Ltd., Shanghai, China) with an inner diameter of 340 μm was connected to the syringe, and employed as the printing nozzle. The printing nozzle was fixed on the Z-axis moving system that controlled the printing height during the EHD printing process. A high-resolution XY moving stage (AI-LM-XY15000AB, Alio Industries, Arvada, CO, USA) was utilized to collect the ejected fibers in a layer-by-layer manner according to user-specific trajectory. The ejected fibers were deposited onto the ITO conductive glass (Guluo Glass Co., Ltd., Luoyang, China) with the with the sheet resistance of 10 Ω/sq. A high voltage supply was connected between the printing nozzle and glass collector, providing the electrical force that initiated and maintained the ejecting process. The working voltage, nozzle-to-collector distance, stage moving speed, and feeding rate were adjusted by the computer. To fabricate the PVDF film, the above key processing parameters were controlled at 1400 V, 3 mm, 50 mm/s, and 40 μL/h.

### 2.3. The Assembly of MoS_2_ Nanostructures on EHD-Printed Film

**Figure 1:**
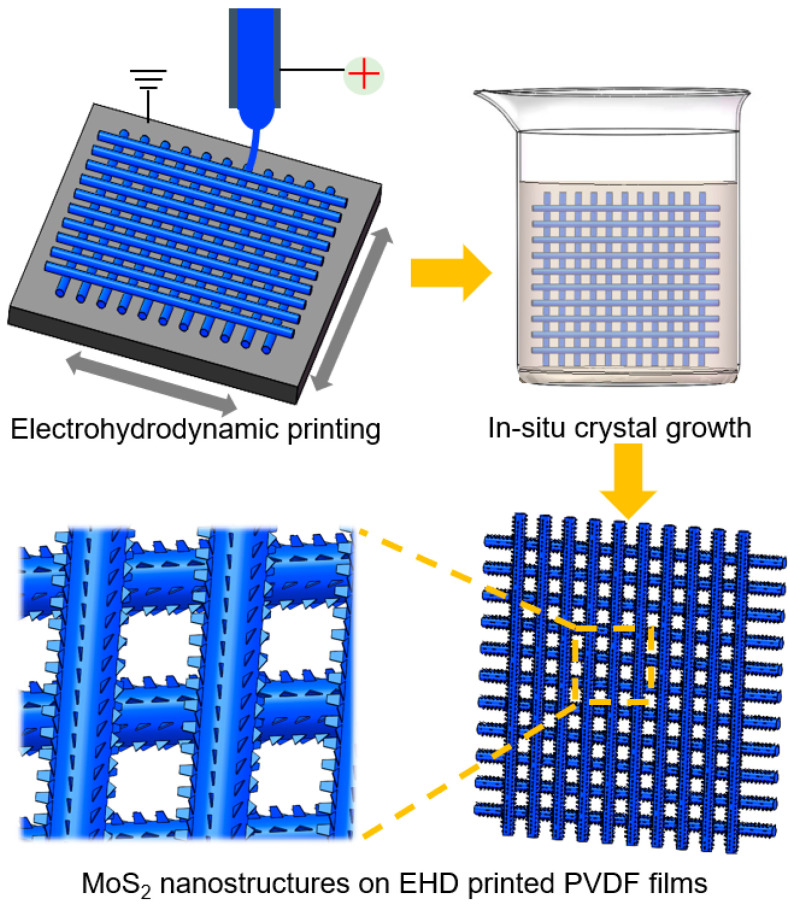
The fabrication process of the MoS_2_-PVDF composite film.

The MoS_2_ nanostructures on PVDF fibers were fabricated by using the hydrothermal crystal growth method. Hexa ammonium heptamolybdate tetrahydrate ((NH_4_)_6_Mo_7_O_24_·4H_2_O) and thiourea (TTA) were utilized as the raw materials to prepare the precursor solution. Firstly, 0.45 g (NH_4_)_6_Mo_7_O_24_·4H_2_O and 0.9 g TTA were thoroughly dissolved in 50 mL of deionized water under room temperature. Secondly, the EHD-printed PVDF film was immersed in the precursor solution. Thirdly, the precursor solution and PVDF film loaded in a Teflon-lined steel autoclave were kept in an electric oven at 200 °C for 5 h. Finally, the MoS_2_-PVDF composite film was washed with DI water and dried in a vacuum at 80 °C for 12 h. The fabrication process of the MoS_2_-PVDF composite film was illustrated in Figure 1.

### 2.4. Structural Observation and Material Characterization

The ejected fibers during the EHD printing process were observed by a real-time monitoring system. An optical camera was fixed onto the printing platform, which was employed to collect the images of the dynamic ejecting process. The collected images were displayed on a screen and can be utilized to observe or adjust the EHD printing process. The optical images of the MoS_2_-PVDF composite film were observed by a laser confocal microscope (OLS4000, Olympus). The micro/nanoscale features of the MoS_2_-PVDF composite film were obtained by a scanning electron microscope (SEM, SU8010, Hitachi, Tokyo, Japan). Apart from the structural observation, X-ray diffraction (XRD, Bruker, D8, Billerica, MA, USA) and Raman spectrometer (HR Evolution) were also employed to demonstrate the existence of MoS_2_ on PVDF films.

### 2.5. Electrochemical Measurements

The cyclic voltammetry (CV) and galvanostatic charge discharge (GCD) performance of the as-fabricated MoS_2_-PVDF composite film was measured to demonstrate the potential energy storage capacity as supercapacitor electrodes. The electrochemical measuring was conducted by using the three-electrode configuration on an electrochemical working station (Reference 600, Gamry, Warminster, PA, USA). A platinum plate and Ag/Cl electrode were utilized as the counter electrode and reference electrode respectively. The MoS_2_-PVDF composite film was cut into 1 cm × 1 cm slices and utilized as the working electrode. The measurement of CV performance was performed in 1 M Na_2_SO_4_ aqueous electrolyte under the potential window between −0.2 V and 0.8 V. The GCD curve was measured with the current density of 0.5 mA/cm^2^, 1.0 mA/cm^2^, 1.5 mA/cm^2^, and 2.0 mA/cm^2^. The mass specific capacitance was obtained by using the following equation:

(1) $$C_{m}=\frac{1}{ms\Delta V}\int I\left(V\right)dV$$

where *C_m_* is the mass specific capacitance, *m* is the mass of the electrode; *s* is the scanning rate; Δ*V* is the potential window; *I* and *V* represent the current and potential in the CV curve.

## 3. Results

### 3.1. EHD Printing of the Microscale PVDF Fibers

**Figure 2:**
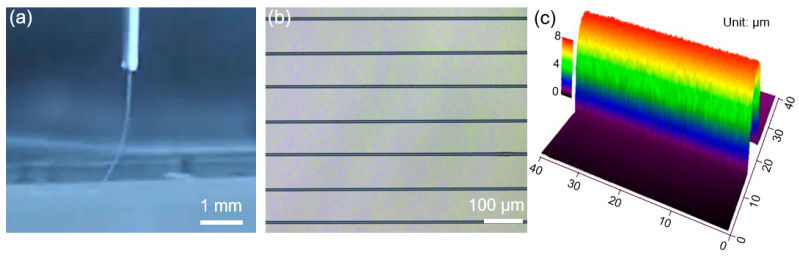
EHD printing of the PVDF fibers. (**a**) The optical image of the ejected fibers from the nozzle, which was obtained by the real-time monitoring system. (**b**) Straight PVDF fibers with the width of 8.3 ± 1.2 μm, with a fiber spacing of 100 μm. (**c**) The 3D profile of a single EHD-printed PVDF fiber.

As shown in Figure 2a, it was observed that the PVDF fibers were formed by the electric force and ejected from the printing nozzle. The fibers were dragged by the moving stage and deposited onto the collector. The moving stage generates a mechanical force on the ejected fiber, which significantly determines the fiber arrangement and direction on the collector. For instance, a lower stage moving speed commonly results in curvilinear fiber arrangement, and a higher stage moving speed normally results in straight fiber morphology. To obtain the straight fibers on the collector, the stage moving speed should be matched with the ejecting speed of the fibers outside the nozzle [18]. In our experiment, the straight PVDF fibers were EHD-printed with a fiber width of 8.3 ± 1.2 μm (Figure 2b). The fiber spacing was tuned at 100 μm according to the programmable trajectory controlled by the computer. The 3D scanning result of the EHD-printed microscale fiber was demonstrated in Figure 2c. The height of the fiber was 7.1 ± 1.5 μm, which was close to the fiber width.

The EHD-printed fiber width is mainly determined by the material’s property and processing parameters. Due to the suitable viscosity and permittivity, PVDF enables well EHD printing stability for microscale fibers [19,20]. The effect of EHD processing parameters on the printed fiber width has been investigated by pioneering studies [21]. Generally, the increased working voltage and feeding rate facilitate the formation of a larger fiber width. The increased nozzle-to-collector distance and stage moving speed result in a decreased fiber width. Apart from the fiber width, the fiber spacing is another important feature to tune the porosity and specific area of the printed structures [22]. EHD printing enables the fabrication of 2D patterns with controlled fiber spacing larger than dozens of microns. However, the adjacent fibers might be disturbed by the electrical force caused by residual charges in the fibers [23,24]. In our previous study, the smallest fiber spacing can be controlled at 10 μm with sub-microscale fiber width [16]. In this study, PVDF fibers were uniformly fabricated under the constant processing parameters of 1400 V, 3 mm, 50 mm/s, and 40 μL/h. The microscale PVDF fibers were utilized as the substrate for the assembly of MoS_2_ nanostructure.

### 3.2. In-Situ Assembly of MoS_2_ Nanostructure on EHD-Printed PVDF Fibrous Films

**Figure 3:**
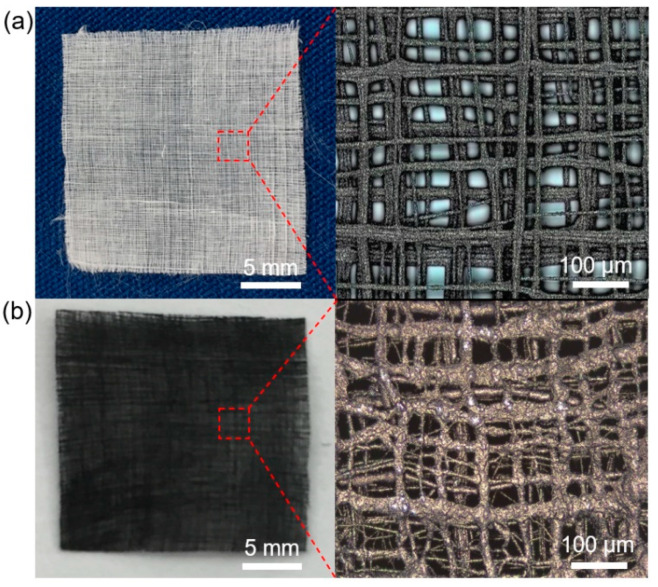
Optical images of the PVDF film and MoS_2_-PVDF composite film. (**a**) Optical images of the PVDF film with the dimension of 2 cm × 2 cm. (**b**) Optical images of the MoS_2_-PVDF composite film.

The microscale fibers were staked in a layer-by-layer manner to form the PVDF film. The EHD-printed 20-layer PVDF film with the dimension of 2 cm × 2 cm is shown in Figure 3a. It was observed that the microscale fibers are distributed vertically and horizontally in the PVDF film. The fibers of different layers were not well-aligned. The malposed position of different layers was caused by the whipping phenomenon as well as the residual charge repulsion between neighboring fibers. In addition, the malposed fibers provide a larger structural surface area than the well-aligned structure, which can facilitate the biological or chemical interactions between EHD-printed structures and the surrounding environments. The PVDF film was further employed as the substrate for the assembly of MoS_2_ nanostructures. Figure 3b shows the optical images of the MoS_2_-PVDF composite film. It was observed that the appearance of the film significantly changed from white to dark, demonstrating the assembly of MoS_2_ on PVDF fibers. Negligible shrinkage was observed on the PVDF fibers which maintained an average size of 8.3 ± 1.2 μm.

**Figure 4:**
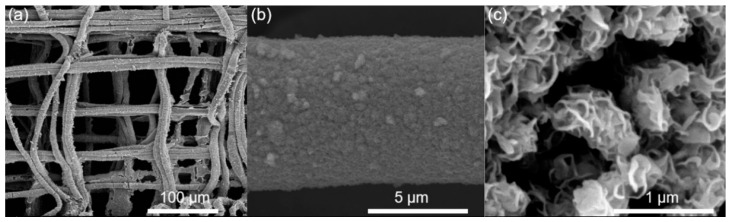
The micro/nanoscale morphology of the MoS_2_-PVDF composite film. (**a**) The microscopic images of the MoS_2_-coated PVDF fibrous film. (**b**) The microscale characterization of the MoS_2_ coated PVDF fiber. (**c**) The SEM image of the MoS_2_ nanostructures.

The micro/nanoscale structural features as well as the materials composition were further characterized to demonstrate the existence of MoS_2_ nanostructures. Figure 4a illustrates the microscale morphology of the MoS_2_-coated PVDF fibrous film. It can be observed that the microscale PVDF fibers maintain the fibrillar shape after the hydrothermal treatment. The MoS_2_ was mainly formed on the surface of PVDF fibers. Negligible MoS_2_ nanostructure was observed in the spacing between the fibers. Concerning a single fiber, it was observed that the MoS_2_ nanostructures were synthesized and assembled on the whole surface of the PVDF fiber (Figure 4b). The nanoscale characterization of the formed MoS_2_ nanostructure is shown in Figure 4c. The MoS_2_ nanostructure showed a flower shape morphology composed of a crowd of nanoplates. The in-situ assembled MoS_2_ nanostructure significantly increased the surface area of the composite structure, which might be meaningful to facilitate the interaction with liquid electrolytes for electrochemical applications [25,26].

**Figure 5:**
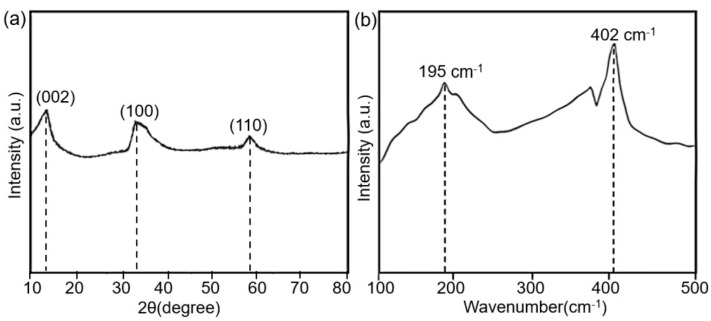
Material characterizations of the synthesized MoS_2_ nanostructures. (**a**) The XRD spectrum of MoS_2_. (**b**) The Raman spectrum of MoS_2_.

The XRD and Raman spectrum were measured and investigated to demonstrate the synthesis of MoS_2_ nanostructures on PVDF fibers. The XRD spectrum of MoS_2_ is shown in Figure 5a. The characteristic peaks at 14.2°, 33.5° and 58.6° in the XRD patterns perfectly matched with the (002), (100), and (110) planes of MoS_2_, respectively. The characteristic peaks are in good agreement with the standard peaks of hexagonal MoS_2_ (JCPDS card No. 37-1492), demonstrating the high-quality crystallization of MoS_2_. The Raman spectrum of MoS_2_ was also investigated, as shown in Figure 5b. The prominent peaks at 195 cm^−1^ and 402^−1^ validate the presence of pristine MoS_2_ on the PVDF fibers. Overall, the XRD as well as the Raman results demonstrated the successful synthesis of MoS_2_ on the EHD-printed fibers.

### 3.3. Potential Energy Storage Capacity of the MoS_2_-PVDF Composite Film as Supercapacitor Electrode

**Figure 6:**
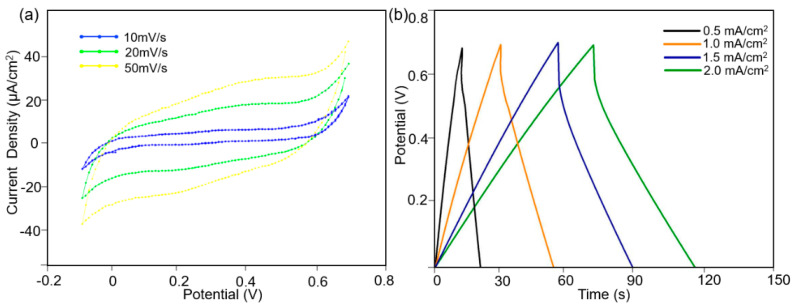
The electrochemical performance of the as-fabricated MoS_2_-PVDF composite electrodes. (**a**) The CV performance measured in the potential widow between −0.2 V and 0.8 V. (**b**) The GCD curve measured with the current density of 0.5, 1, 1.5, and 2 mA/cm^2^, respectively.

**Figure 7:**
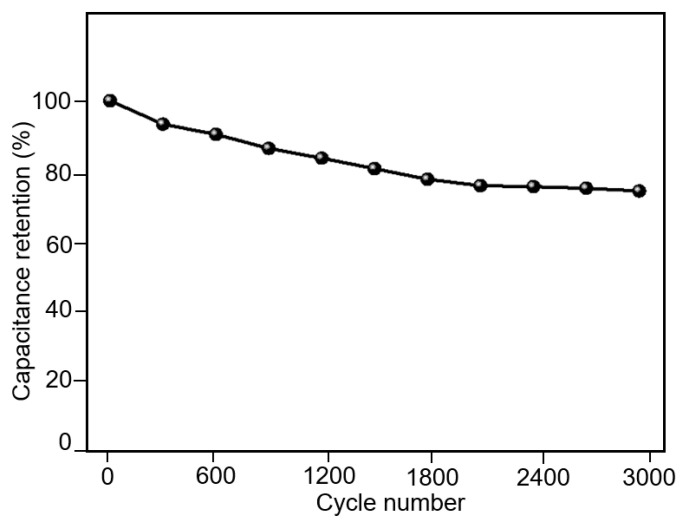
The electrochemical stability of the MoS_2_-PVDF composite electrodes, under the scanning rate of 50 mV/s.

To explore the potential energy storage capacity, the as-fabricated MoS_2_-PVDF composite film was further employed as supercapacitor electrodes for electrochemical investigation. The cyclic voltammetry (CV) measurement is widely utilized to investigate the energy storage applications of supercapacitors. During the CV measurement, a potentiostat is used to linearly sweep the potential between the working and reference electrodes until it reaches the defined limit, at which point it sweeps back in the opposite direction. The CV performance of the MoS_2_-PVDF composite electrode in the potential widow between −0.2 V and 0.8 V is shown in Figure 6a. The CV performance of the MoS_2_-PVDF composite films was measured under the scanning rate of 10 mV/s, 20 mV/s, and 50 mV/s. The typical enclosing curves with no distinctive peaks were obtained under a scanning cycle, indicating the potential charge storage capacity of electrical double-layer capacitance [27,28]. It was calculated that the mass specific capacitance of the electrodes was 60.2 ± 4.5 F/g. The galvanostatic charge discharge (GCD) result was also measured under the current density of 0.5, 1, 1.5, 2 mA/cm^2^. As shown in Figure 6b, the GCD curves exhibited a typical triangular profile that represent the practical capacitance for supercapacitors. The extensive cycling was performed to demonstrate the cyclic stability of the electrode. After 3000 cycles of scanning, the electrodes retained 76.5% of the initial capacitance, as shown in Figure 7.

## 4. Discussion

This work provides a promising strategy to fabricate MoS_2_-PVDF composite electrode for potential energy storage applications. By using the EHD printing process, microscale PVDF films were fabricated with uniform fiber width and controlled fiber arrangement. The MoS_2_ nanostructures were assembled via hydrothermal crystal growth. This work offers an innovative approach for the fabrication of high-resolution and multi-dimensional energy storage electrodes. (i) In the fields of additive manufacturing, various 3D printing techniques have been explored for the fabrication of energy storage electrodes and even devices [29]. Most of the existing 3D-printed electrodes have a manufacturing resolution of larger than dozens of microns. The EHD-printed electrodes significantly improved the manufacturing resolution compared with traditional 3D printing techniques like extrusion-based 3D printing [30] and fused deposition modeling [31]. (ii) On the other hand, the conventional 3D printed electrodes are mainly limited to a single microscale dimension. The assembly of MoS_2_ nanostructures on PVDF fibers provides a promising approach to enrich the structural topology for enhanced electrochemical performance [32].

The proposed MoS_2_-PVDF composite electrodes might also provide innovative inspirations for high-performance energy storage. Electrochemical energy storage involves manifold interactions between the electrode and the surrounding electrolyte [33]. The as-fabricate MoS_2_-PVDF composite electrodes significantly increased the electrode-electrolyte contact interface, which can promote ion transmission as well as electron transfer during the charge-discharge process [34]. PVDF was employed because of its excellent EHD printing performance. However, the low conductivity of PVDF restrains the energy storge performance. Although the specific capacitance of the MoS_2_-PVDF composite electrode remains a relatively low level, the energy storage capacity might be improved by using a high-conductivity current collector or coating conductive materials [35,36]. The EHD printing of high-conductivity materials is still the main challenges due to the electric-induced breakage of the ejected fibers. Future breakthroughs might be conducted in the aspect of innovative printing nozzle configuration that maintains the high voltage to drive EHD ejecting but restrains the internal current within the fibers.

This work preliminarily explores the electrochemical performance and demonstrates the promising energy storage capacity of the MoS_2_-PVDF composite electrodes. It should be mentioned that the fiber density in the EHD-printed electrodes can affect the capacitance for energy storage. By adjusting the fiber spacing, the porosity as well as the surface area can be further optimized for improved electrochemical performance. In our previous study, the structural porosity of the EHD-printed carbon-nickel electrode was tuned from in a wide range of 13.2 ± 4.6 % to 73.6 ± 6.9 % by simply changing the user-specific fiber spacing [17]. Apart from structural control and optimization, the EHD printing process also enables the microminiaturization of energy storage devices [37]. As a large majority of the existing energy storage materials were synthesized by chemical methods, which were not able to directly customize the geometrical shape of the electrodes. The proposed method for MoS_2_-PVDF composite electrodes can facilitate the fabrication of tiny energy storage devices with pre-designed morphology and high resolution. In addition, the employed PVDF possesses advanced functions like piezoelectric property and flexibility, which may be further explored for flexible energy generation and storage devices [38,39].

## 5. Conclusions

This work presented a novel strategy to fabricate multidimensional electrodes involving microscale PVDF fibers and MoS_2_ nanostructures. EHD printing was developed to create the microscale PVDF film with a stable fiber width of 8.3 ± 1.2 μm. Combining with the hydrothermal crystal growth method, MoS_2_ nanostructures were synthesized and assembled on the microscale PVDF fibers. The PVDF films maintained a stable morphology after the high-temperature treatment. Microscopic observations as well as material characterizations were conducted to demonstrate the existence of MoS_2_ on the PVDF fibers. It was observed that the as-fabricated MoS_2_-PVDF electrodes showed a typical charge-discharge curve during the electrochemical experiments. The proposed method enriched the structural and dimensional properties of 3D printed electrodes, which might be further explored for the optimization and micro miniaturization of energy storage devices.
